# Text difficulty modulates the surprisal effect in self-paced reading

**DOI:** 10.1007/s11145-026-10768-7

**Published:** 2026-02-11

**Authors:** Lin Chen, Gaisha Oralova, Xiaoping Fang, Shannon Clark, Daniela Teodorescu, Maxwell Helfrich, Alona Fyshe, Carrie Demmans Epp, Charles Perfetti

**Affiliations:** 1 University of Illinois Urbana-Champaign, Champaign, USA; 2 University of Pittsburgh, Pittsburgh, USA; 3 Beijing Language and Culture University, Beijing, China; 4 University of Alberta, Edmonton, Canada

**Keywords:** Word surprisal, Syntactic surprisal, Text difficulty, Reading

## Abstract

Surprisal, the statistical likelihood of a word or syntactic structure given its preceding context, is an important metric for understanding the predictive/integrative process in reading. Although individual words and syntactic structures vary in surprisal within any text, regardless of its overall difficulty, it remains unclear whether text difficulty modulates how surprisal influences reading. We hypothesized that higher text difficulty increases readers’ reliance on contextual information during reading, leading to stronger surprisal effects. To test this hypothesis, we examined how text difficulty modulates the strength of word and syntactic surprisal effects and whether this modulation generalizes across computational language models with different architectures. We compared reading times for two sets of texts that differed in difficulty but were matched on average surprisal. Results showed larger word and syntactic surprisal effects in more difficult texts, consistently across models. These results suggest that the predictive/integrative processes are shaped by global text properties.

## Introduction

During reading, readers incrementally and continuously integrate individual words into the previously read context to construct a coherent mental representation of the text (Baggio & Hagoort, 2011; Chen et al., 2017; Chen et al., 2025; Perfetti & Stafura, 2014), a process that can be enhanced by predictive mechanisms (DeLong et al., 2005; Kuperberg & Jaeger, 2016). These *predictive/integrative* processes are fundamental to meaning construction in fluent reading. A key challenge in understanding how these processes unfold is to assess how well each word in a text aligns with its preceding context and how this alignment affects reading behavior.

### Surprisal and reading processes

A recently prominent computational measure used to quantify this contextual alignment is *surprisal* (Hale, 2016; Levy, 2008), defined as the negative logarithm of the probability of the word given the preceding context (see the following formula). This measure is computed using probabilistic language models trained on text corpora, where words that are less probable in a given context receive higher surprisal values.

$$ S(w_{t + 1} ) = - logP(w_{t + 1} |w_{1 \ldots t} ) $$

Surprisal can be computed at different linguistic levels, including the word level and the syntactic (part-of-speech) level. *Word surprisal* quantifies the probability of a specific word given the preceding text, thus incorporating both semantic and (implicitly) syntactic information. In contrast, *syntactic surprisal* estimates the probability of a word’s syntactic category (part-of-speech) based on the syntactic category information of preceding words in a sentence, capturing only structural information independent of semantic information. These two measures capture distinct sources of linguistic information, enabling assessment of their respective contributions to meaning construction (Demberg & Keller, 2008; Frank et al., 2015; Roark et al., 2009). Comparing them also provides insights into readers’ sensitivity to syntactic versus semantic information and helps clarify the long-standing debate about the separation of syntactic and semantic processing (Fedorenko et al., 2012, 2020; Friederici, 2011; Keller et al., 2001; MacDonald et al., 1994; Pylkkänen, 2019). For example, Arehalli et al. (2022) found that including syntactic surprisal as a predictor in linear mixed-effects models improved the prediction of human reading times for garden path sentences compared to models that included only word surprisal. This highlights the value of syntactic surprisal in capturing the distinct cognitive demands associated with syntactic processing.

Empirical evidence shows that surprisal predicts both behavioral and neural measures of reading (Chen et al., 2025; Fernández Monsalve et al., 2012; Frank et al., 2015; Oralova et al., 2026; Roark et al., 2009). Words with higher surprisal values are associated with longer reading times (Fernández Monsalve et al., 2012; Roark et al., 2009) and more negative N400 amplitudes (Chen et al., 2025; Frank et al., 2015; Oralova et al., 2026), indicating increased cognitive effort when the current word is less consistent with its preceding context. However, there remains debate about which specific cognitive processes these surprisal effects reflect. Because surprisal quantifies how unexpected a word is given its prior context—the inverse of word predictability—it has often been interpreted as an index of predictive processing during reading (Cevoli et al., 2022). At the same time, surprisal effects are frequently observed on relatively late neural measures, such as the N400 (Chen et al., 2025; Frank et al., 2015; Oralova et al., 2026) and the P600 (Wang et al., 2026), suggesting that surprisal may also reflect post-lexical integration processes that occur after a word is encountered rather than solely anticipatory prediction (see Wong et al., 2025, for a detailed discussion of these competing interpretations). Recent studies, therefore, interpret surprisal as reflecting integrative processes that are facilitated by prediction, often referred to as predictive/integrative processes (Chen et al., 2025; Oralova et al., 2026). Under this view, words of higher surprisal require greater cognitive effort because the preceding context provides less reliable cues for predicting and integrating the upcoming word into the reader’s mental representation of the text.

Although surprisal is a valuable tool for examining reading processes, the strength of its effects may be influenced by text features that are not captured by the surprisal measure itself. Surprisal reflects the alignment between a word and its preceding context, and any text, regardless of its overall difficulty, will show variation in surprisal across its individual words and syntactic categories (i.e., word surprisal and syntactic surprisal). However, this variation may not influence readers uniformly across different levels of text difficulty. Text difficulty is an aggregate property encompassing multiple linguistic dimensions, including lexical, syntactic, and discourse features. These dimensions collectively shape cognitive demands during reading, and, in turn, may modulate the magnitude of surprisal effects.

### Reading processes adjust to text difficulty

Readers adapt their reading processes based on the difficulty of the text (Alter, 2013). For example, when reading more difficult texts, readers may experience cohesion gaps–breaks in the explicit connections between ideas (McNamara, 2001), prompting greater allocation of cognitive resources for comprehension. More generally, readers may adapt to more challenging texts by investing more time and effort (Fulmer & Frijters, 2011; Fulmer et al., 2015) and by engaging in deeper cognitive processes (Alter, 2013), e.g., making elaborations and inferences, to support meaning integration and maintain coherence (Albrecht & O’Brien, 1993; Linderholm et al., 2000). These adaptive strategies generally increase the active involvement of memory, leading to better learning and retention of the text content (Bjork & Bjork, 2011) (Extremely challenging texts are beyond the scope of this paper because readers may experience breakdowns in comprehension processes while reading them (Britton et al., 1978)).

Specifically important to the effects of surprisal on word reading is the possibility that reading difficult texts prompts a greater reliance on context compared to easier texts. Difficult texts often include low-frequency words and complex structures, whereas easier texts typically contain high-frequency words and simpler syntax (Graesser et al., 2004). Thus, easier texts allow fluent word identification, meaning retrieval, and sentence parsing—processes that require more cognitive resources in more challenging texts. To manage these demands, readers may rely more heavily on contextual information, using it to support both lexical and syntactic processing. Context, for example, can help readers identify unfamiliar words and retrieve their meaning (Becker, 1979) and can also facilitate syntactic parsing by pre-activating a word’s grammatical role within a sentence (Matar et al., 2019). Moreover, reading difficult texts requires active engagement with memory (Bjork & Bjork, 2011), which allows readers to draw on prior context more effectively to construct meaning. This increased reliance on context may serve a compensatory function, helping readers manage the heightened cognitive demands imposed by difficult texts and thereby support comprehension.

If our assumption is correct, that reading difficult texts requires a greater reliance on context, then text difficulty should modulate the effect size of surprisal, which quantifies how well a word aligns with its context. In other words, a greater reliance on context in difficult texts should enhance the predictive power of surprisal, leading to larger surprisal effects on reading measures compared with easier texts.

### Generalizability of the effect of text difficulty on surprisal across computational language model architectures

Although surprisal is defined by the same mathematical formula across computational language models, each model estimates the conditional probability of a word given its preceding context differently, depending on how linguistic structure is represented. Therefore, when testing the effect of text difficulty on surprisal, it is critical to include models that differ in their representational assumptions to ensure generalizability.

Previous research has shown that surprisal estimates derived from different model architectures vary in how well they capture human reading behavior, although findings have been mixed (Merkx & Frank, 2020; Oh et al., 2022; Oralova et al., 2026). For example, surprisal derived from Transformer-based models has been shown to outperform that from Recurrent Neural Network (RNN) models in predicting human reading times, even when both models were trained on the same dataset (Merkx & Frank, 2020). In contrast, other research has found that simpler models, such as Probabilistic Context-Free Grammar (PCFG) models, can outperform Transformer-based models like GPT-2 in predicting reading times (Oh et al., 2022), suggesting that stronger computational performance does not necessarily imply closer alignment with human reading processes, which are shaped by cognitive constraints such as limited working memory.

Given that computational language models differ in how they represent linguistic structure and generate surprisal estimates, examining whether the modulation of surprisal by text difficulty generalizes across models provides a stronger test of the robustness of this effect. To this end, we used four language models with distinct architectural frameworks: N-gram, Probabilistic Context-Free Grammar (PCFG), Recurrent Neural Network Grammar (RNNG), and Transformer models, ranging from simpler to more advanced architectures, to assess the generalizability of the text difficulty on surprisal.

### The present study

This study investigates how text difficulty modulates surprisal effects during reading and whether this modulation generalizes across computational language models with distinct architectures. Specifically, we address two questions: (1) Does text difficulty modulate surprisal effects consistently across different types of surprisal, namely, word surprisal and syntactic surprisal, during reading? (2) Do these effects generalize across computational language models that differ in their representational architectures?

To answer these two questions, we compared reading behaviors of easy and difficult texts that were comparable in average surprisal values across all words in the text but differed in overall text difficulty. This design isolates the moderating role of text difficulty while controlling for surprisal, allowing us to test how readers adapt to overall text difficulty beyond what can be explained by the surprisal value of individual words during comprehension.

To address the first question, we adopted reading materials from Chen et al. (2025) and Oralova et al. (2026), which investigated surprisal effects using EEG. The texts were organized into two groups based on difficulty and evaluated using three metrics: word frequency (lexical difficulty), syntactic complexity (syntactic difficulty), and overall readability (overall difficulty). One group of texts is defined as “easy” and the other group as “difficult”. The easy texts scored lower on all three difficulty measures compared to the difficult texts, while the two sets were matched on topic familiarity. We computed two types of surprisal, word surprisal and syntactic surprisal, and tested the effects of text difficulty on each type. Word surprisal reflects both semantic and syntactic information from the preceding context, whereas syntactic surprisal indicates how well a word’s syntactic category aligns with the prior structural context.

We predicted that text difficulty would enhance surprisal effects across both word and syntactic surprisal. As comprehension becomes more cognitively demanding in difficult texts, readers are expected to rely more heavily on contextual information to process words and facilitate comprehension. This increased reliance on context should heighten sensitivity to how well a word aligns with its preceding context, producing stronger surprisal effects than in easier texts.

To address the second question, we examined surprisal effects using four computational language models: N-gram, PCFG, RNNG, and Transformer models, ranging from simple statistical to advanced neural architectures, to evaluate whether the modulation of surprisal by text difficulty is robust across model architectures. Moreover, because they vary in how they represent syntactic information, some explicitly, others implicitly, they are particularly well-suited for testing whether the modulation of text difficulty on syntactic surprisal effect remains consistent across models (For details on model architecture, training procedures, and surprisal computation, see section “Surprisal and computational language model training” and Table 2).

## Method

### Participants

Thirty-four (average age = 18.7, *SD* = 2; 30 female) and thirty-five (average age = 18.5, *SD* = 0.95; 28 female) native English readers from the University of Pittsburgh participated in the study by reading the easy and more difficult texts, respectively, with course credit provided as compensation. The between-subject design was adopted to help maintain participants’ engagement, as extended reading sessions in a laboratory setting may lead to reduced attentiveness. Data from one participant in the easy text condition were excluded from the data analysis due to a procedural error during data collection. All participants were right-handed with normal or corrected-to-normal vision and without any history of head injury or neurological diseases. Each participant provided informed consent before participating in the experiments.

To ensure that the two participant groups had comparable English reading proficiency, we administered the Nelson-Denny vocabulary and comprehension tests (Nelson & Denny, 1973). The vocabulary test consists of 100 multiple-choice items to complete in 7.5 min, while the reading comprehension test includes eight passages and 36 multiple-choice questions to complete in 15 min. Scores were calculated as the percentage of the correct answers on each test. For participants who read the easy texts, the average vocabulary and comprehension scores were 59.21 (*SD* = 11.53) and 67.76 (*SD* = 13.19), respectively. For those who read the difficult texts, the corresponding scores were 55.66 (*SD* = 16.64) and 72.75 (*SD* = 14.30). The two groups did not show differences in either the vocabulary test (*t*_(61)_ = − 1.03, *p* = 0.31, Cohen’s *d* = − 0.25) or the comprehension test (*t*_(66)_ = 1.50, *p* = 0.14, Cohen’s* d* = 0.36).

### Stimuli

The stimuli included two sets of texts, differing in text difficulty, both extracted from *the New York Times*. Each passage consisted of the first two sentences from articles published in 2018 and 2019 (see Appendix for the complete list). Using the first two sentences of an article allows the reader to understand the text without requiring any prior context. With two sentences in each passage allowing a continuous range of values of variables, we are also able to examine the reading processes across a sentence boundary. The passages were sampled from a broader range of New York Times categories, including Arts, Business, Food, Health, New York, Opinion, Parenting, Science, Style, Travel, U.S., and World. We excluded articles describing high-profile current events that would receive widespread media coverage and articles with highly culture-specific topics to minimize the influence of background knowledge or media exposure and to maintain a focus on core reading processes.

Both the easy and difficult text sets contained 63 two-sentence passages, 20 of which were shared across groups to ensure comparability between participants in this between-subject design (See Table 1). For data analysis, we focused on the 43 passages that were unique to each group, which provided the critical contrast for testing how text difficulty modulates the surprisal effect.

**Table 1:** Measures of text difficulty

	Mean word frequency (*SD*)	Syntactic complexity		Mean readability (*SD*)
		Mean number of words before the main verb (*SD*)	Mean number of modifiers per noun phrase (*SD*)	
Easy texts	4.26 (*1.40*)	2.98 (*2.21*)	0.75 (*0.35*)	7.11 (*2.83*)
Difficult texts	4.06 (*1.55*)	4.47 (*2.59*)	0.90 (*0.30*)	8.76 (*2.40*)
Shared texts*	4.33 (*1.36*)	2.98 (*1.67*)	0.79 (0*.44*)	6.96 (*2.22*)

*The 20 shared passages were designed to ensure participant comparability, and analyses focused on the 43 unique passages per group

We define text difficulty through three dimensions: lexical difficulty, syntactic difficulty, and overall readability. We used word frequency as an estimate of lexical difficulty. Typically, difficult texts contain a higher proportion of low-frequency words that are less familiar to readers and whose meanings may be more difficult to retrieve (Ratcliff et al., 2004; Rayner & Duffy, 1986). In contrast, easier texts tend to have more high-frequency words that readers encounter frequently and for which they have more robust representations (Perfetti, 2007); thus, those words can be processed quickly. Word frequency was obtained from the SubtlexUS dataset (Brysbaert & New, 2009).

We used syntactic complexity to measure syntactic difficulty. Difficult texts typically have more complex syntactic structures (Graesser et al., 2004), requiring longer reading times than texts with simpler structures (Gibson, 1998). For example, when reading garden path sentences, readers often experience a slowdown in reading times (Christianson et al., 2006) due to the temporary ambiguity in the syntactic structure of such sentences. Given the critical roles of head verbs and head nouns in determining syntactic structure and meaning interpretation, we assessed syntactic complexity using two Coh-Metrix measures (Graesser et al., 2011): the number of words before the main verb and the number of modifiers per noun phrase.

Readability measures are commonly used metrics to indicate overall text difficulty (Klare, 1974). One widely used measure is the Flesch-Kincaid Grade Level, which estimates how easily a text can be read by measuring coarse-grained lexical and syntactic properties of sentences. It calculates readability based on the average number of syllables per word and the average sentence length in the text being evaluated. Words with more syllables, typically of lower frequency, require greater effort and result in longer reading times. Similarly, longer sentences generally reflect greater syntactic complexity, increasing the effort needed for comprehension. We used the Flesch-Kincaid Grade level (Kincaid et al., 1975) as implemented in Coh-Metrix to assess readability.

The two groups of texts are different in all text difficulty measures: Word frequency, *t*_(2108)_ = **− **3.13, *p* < 0.01; Number of words before the main verb, *t*_(82)_ = 2.86, *p* < 0.01; The number of modifiers per noun phrase, *t*_(82)_ = 2.14, *p* = 0.035; Flesch-Kincaid Grade Level, *t*_(82)_ = 2.91, *p* < 0.01. Details of the results for the three measures in each group are presented in Table 1.

Eighty-three additional participants (average age = 19.08, *SD* = 1.71, 53 female), drawn from the same college student population as those in the main experiment, rated their familiarity with the topics of the two text sets using a 7-point Likert scale (1 = very unfamiliar, 7 = very familiar). The ratings indicated that the topics of the two text sets were similarly familiar to the subjects: *Mean* = 4.87 (*SD* = 0.93) and *Mean* = 4.75 (*SD* = 0.87).

### Procedure

#### Procedure of self-paced reading

The stimuli were presented using PCIbex (Zehr & Schwarz, 2018) in a self-paced moving window paradigm. Each passage was initially presented as a series of dashes that corresponded to the number of letters in each word, which were then replaced by the letters of the word. Participants were instructed to read at their natural pace and to make sure they understood what they had read. Pressing the spacebar revealed the next word, replacing the dashes. Reading times for each word were recorded as the interval between keypresses. Words were presented in black on a white background. Commas and periods were presented with the preceding word. The font size was 20.

Two-thirds of the passages were followed by a true/false comprehension question, with half of the correct answer being “true.” These questions were designed to assess overall passage understanding by targeting main ideas and essential details. While not especially difficult, they required careful reading, reinforcing engagement, and serving as a reliable measure of comprehension. For example, consider the following passage: *“A number of countries, including Australia and Germany, have started requiring companies to report their gender pay gaps in recent years. Proponents argue that transparency can lead to improvement.”* The corresponding comprehension question asks readers to judge whether the following statement is true or false: “*In Germany, companies must report gender pay gaps in hopes of improvement.”*

The question appeared immediately after the offset of the last word of the passage. Participants pressed a designated key to indicate their answers and received feedback afterward. (If they did not answer the question within 10 s, they were given a reminder.) After the feedback, participants pressed the spacebar to start the next trial. After reading each passage, participants could take a break or move to the next passage. To encourage engagement during reading, participants received a reminder if they failed to press the spacebar within 30 s. The passages were presented randomly following six practice items, four of which included comprehension questions. The experiment lasted less than 30 min.

#### Surprisal and computational language model training

We used word and syntactic surprisal measures calculated by four types of computational language models: PCFG, N-gram, RNNG, and Transformer. Word surprisal calculates the probability of a word based on the preceding words in the sentence. Syntactic surprisal calculates the probability of a word’s part-of-speech (POS) based on the POS of preceding words and other syntactic information, which varies depending on the specific language model employed.

These models differ in their architectural frameworks and in how they represent linguistic structures, allowing us to examine whether text difficulty modulates word and syntactic surprisal effects consistently across models. N-gram and PCFG are simple statistical models: the N-gram model captures local word co-occurrence patterns, while the PCFG model applies predefined probabilistic phrase structure rules. RNNG and Transformer are neural network-based models capable of learning more complex linguistic features, including long-range dependencies. Importantly, the models also differ in how they represent syntax. PCFG and RNNG explicitly encode hierarchical structure—PCFG through rule-based probabilistic grammars and RNNG through learned recursive compositions using recurrent neural networks. In contrast, N-gram and Transformer models implicitly infer syntactic structure from word sequences: N-gram through local statistical associations and Transformer through parallel attention mechanisms that capture word dependencies without explicit syntactic parsing (Table 2).

**Table 2:** Comparisons of language model architectures

Models	Architecture	Strength	Features of the training dataset
N-gram	Statistical	Local word patterns	Sequence of words
Transformer	Neural network	Abstract representations	Sequence of words
PCFG	Statistical	Predefined grammatical rules	Hierarchical syntactic structure
RNNG	Neural network	Abstract syntactic representations	Hierarchical syntactic structure

All language models were trained on the WikiText-2 raw dataset (Merity et al., 2016). The dataset includes 720 articles, with 600 allocated for training and 60 each for validation and testing. We selected this dataset because its structured content closely matches the register of our experimental materials. This linguistic and stylistic similarity is important for generating accurate surprisal estimates (Hale et al., 2019).

The different nature of the language models used required datasets with different formats to enable training and the calculation of stimulus surprisal. After pre-processing the raw WikiText-2 dataset, we prepared four versions of the data for model training: words, POS tags of words, lexicalized trees, and unlexicalized trees. POS tags were generated using spaCy (Honnibal & Montani, 2018). To construct the lexicalized trees for the WikiText-2 dataset, we used Stanford Parser 4.2.0 (Klein & Manning, 2003). Unlexicalized trees were obtained by replacing the words in the lexicalized trees with their POS tags. The detailed training procedure for each type of model is outlined below:

*PCFG*: We used an incremental top-down parser (Roark, 2001; Roark et al., 2009) to acquire phrase structure trees for training PCFG language models. Two PCFG models were trained as described in Demberg and Keller (2008): one on lexicalized trees containing terminal words and the other on unlexicalized trees without terminal words. These models were used to compute word surprisal and syntactic surprisal, respectively. A threshold of 0.02 was selected for both the lexicalized and unlexicalized trees through hyperparameter tuning that aimed to minimize the word perplexity.

*N-gram*: We trained two N-gram models using KenLM (Heafield, 2011), one on the dataset of words and another on the dataset of POS tags. These models were used to compute word surprisal and syntactic surprisal, respectively. After hyperparameter tuning, the optimal value of ‘n’ was determined to be 6 for the model to compute word surprisal and 5 for the model to compute syntactic surprisal. These values were selected because they minimized perplexity on the validation set.

*RNNG*: We trained two RNNG models, one on lexicalized trees and another on unlexicalized trees, using the PyTorch implementation (Noji & Oseki, 2021) to obtain word surprisal and syntactic surprisal. The final hyperparameter included a learning rate of 0.002, a dropout rate of 0.2, a batch size of 256, a batch composition consisting of sentences with random lengths, and a beam size of 400. After hyperparameter tuning, the RNNG employed a two-layer long short-term memory (LSTM) network with 256 hidden dimensions.

*Transformer*: We trained two two-layer transformer models. We used the words dataset to train one model and the POS tags to train the other, following Merkx and Frank (2020). After parameter tuning, we chose a learning rate of 0.005, a dropout rate of 0.1, and a batch size of 5. The model included an embedding layer, where each word is represented by a 400-dimensional vector, two transformer layers, with eight attention heads each, and a fully connected layer with 1024 units.

The average surprisal values across all words are comparable for the easy and difficult texts (See Table 3). Welch’s t-tests showed no significant differences between easy and difficult texts in either word surprisal or syntactic surprisal across four models: PCFG (word surprisal: *t*_(2087)_ = 0.74, *p* = 0.46; syntactic surprisal: *t*_(2001)_ = − 0.57, *p* = 0.57); N-gram (word surprisal: *t*_(2063)_ = 1.27, *p* = 0.20; syntactic surprisal: *t*_(2057)_ = − 0.13, *p* = 0.90); RNNG (word surprisal: *t*_(2123)_ = 1.84, *p* = 0.07; syntactic surprisal: *t*_(2003)_ = − 0.80, *p* = 0.42); Transformer (word surprisal: *t*_(2090)_ = 1.73, *p* = 0.08; syntactic surprisal: *t*_(2025)_ = − 0.23, *p* = 0.82).

**Table 3:** Surprisal values calculated from different language models

Model	Surprisal	Easy texts			Difficult texts		
		Range	Mean	SD	Range	Mean	SD
PCFG	Word Surprisal	0.41–17.65	6.90	3.43	0.30–19.83	7.02	3.69
	Syntactic Surprisal	0.02–10.11	2.38	1.49	0.02–8.94	2.34	1.48
N-gram	Word Surprisal	0.001–15.50	7.08	3.71	0.007–15.85	7.28	3.89
	Syntactic Surprisal	0.002–7.42	2.33	1.44	0.006–9.15	2.33	1.51
RNNG	Word Surprisal	0.002–27.16	6.81	4.06	0.02–26.75	7.15	4.58
	Syntactic Surprisal	0.01–8.46	2.25	1.48	0.02–8.98	2.20	1.47
Transformer	Word Surprisal	0.003–16.58	6.85	3.81	0.005–16.64	7.14	4.11
	Syntactic Surprisal	0.007–8.03	2.26	1.49	0.007–9.50	2.24	1.50

**Fig. 1:**
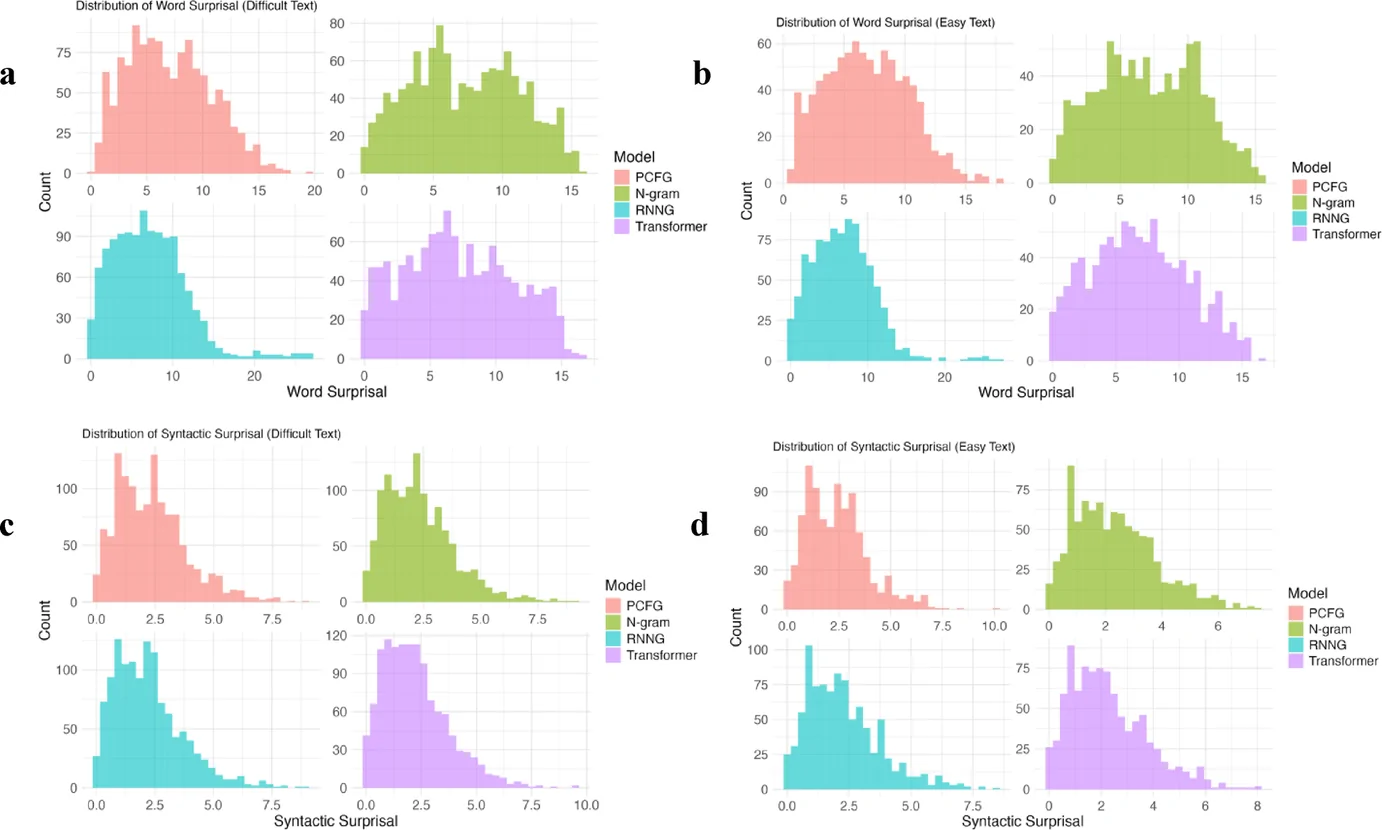
Distribution of surprisal values. **a** Distribution of word surprisal in the difficult texts. **b** Distribution of word surprisal in the easy texts. **c** Distribution of syntactic surprisal in the difficult texts. **d** Distribution of syntactic surprisal in the easy texts

Beyond these mean comparisons, we also examined the full surprisal distributions to ensure broader comparability across conditions. The distributions were generally similar across models, with only marginal differences observed in word-level surprisal for the RNNG and Transformer models (both *p*s ≈ 0.045), suggesting that variance in surprisal was largely matched between easy and difficult texts. Distribution plots for each model and the results of Kolmogorov–Smirnov tests comparing surprisal distributions across text difficulty levels are in Fig. 1 and Appendix Table 8.

The surprisal values, for both word and syntactic surprisal, were highly correlated across the four language models (See Tables 4 and 5), indicating that the models yield comparable surprisal estimates.

**Table 4:** Word surprisal correlations among four models

	PCFG	N-gram	RNNG	Transformer
PCFG		**0.87**	**0.75**	**0.85**
N-gram	*0.89*		**0.87**	**0.91**
RNNG	*0.80*	*0.87*		**0.89**
Transformer	*0.87*	*0.90*	*0.90*	

Values in the upper triangle indicate correlations for difficult texts, while values in the lower triangle correspond to easier texts. All correlations are significant at *p* < 0.001

**Table 5:** Syntactic surprisal correlations among four models

	PCFG	N-gram	RNNG	Transformer
PCFG		**0.92**	**0.93**	**0.93**
N-gram	*0.89*		**0.90**	**0.90**
RNNG	*0.94*	*0.88*		**0.96**
Transformer	*0.93*	*0.90*	*0.96*	

Values in the upper triangle indicate correlations for difficult texts, while values in the lower triangle correspond to easier texts. All correlations are significant at *p* < 0.001

#### Data analysis

During data analysis, passages for which participants answered the comprehension questions incorrectly were excluded. Further, we excluded words whose reading times were longer than 3000 ms or shorter than 100 ms (Wehbe et al., 2021), as well as those beyond 2.5 *SD* of a participant’s average reading time (Tremblay et al., 2011). As a result, 6% and 11% of the data were excluded in the easy and difficult groups, respectively. In data analyses, we excluded the first and last words to avoid their extreme influences as “start-up effects” and “wrap-up effects” (Kuperman et al., 2010; Warren et al., 2009). This exclusion also benefits cross-study comparisons with previous work, which analyzed only middle words (Frank et al., 2015; Roark et al., 2009).

We combined data from both text difficulty groups into a single linear mixed-effects (LME) model to examine how text difficulty modulates the surprisal effect during reading. Given that we have four types of language models, and each model estimated two types of surprisal, we ran eight statistical models in total to test how text difficulty modulates surprisal effects.

In each statistical model, the dependent variable was the reading time for each word. Fixed effects included text difficulty, current-word surprisal, previous-word surprisal, and their interactions with text difficulty. Text difficulty was operationalized as a categorical variable with two levels (easy vs. difficult). The surprisal of the previous word was included to capture potential spillover effects on the current word (Kliegl et al., 2006).

We included word frequency and word length for each word and the word that immediately preceded it because these variables are robust predictors of reading times (Kuperman et al., 2018). We also included position factors as covariates, further including word position (i.e., the position of each word within the sentence), sentence position (whether the word appeared in the first or second sentence of the passage), and their interaction. These positional factors were added because, although they do not reflect specific linguistic properties, they may serve as indicators of the amount of accumulated contextual information as the reading progresses (Kuperman et al., 2018). This accumulated context extends beyond what is captured by surprisal and has been shown to influence reading measures (Chen et al., 2025). Word position was treated as a continuous variable based on a word’s order within a sentence. Sentence position within the text was coded as sentence 1 or 2. Subjects, passages, and words were included as random factors. While a maximal random-effects structure is recommended to ensure generalization and control for Type I error (Barr et al., 2013), we adopted a more parsimonious random-effects structure by including random intercepts only and excluding random slopes for the fixed-effect predictors. This decision was made to avoid singular fit issues and ensure model stability. The final model structure is shown below.

Model<-lmer (RT~ 1 + Surprisal + PreviousWord_Surprisal + TextDifficulty + Surprisal:TextDifficulty + PreviousWord_Surprisal:TextDifficulty + WordFrequency + WordLength + PreviousWord_WordFrequency + PreviousWord_WordLength + WordPosition + SentencePosition + WordFrequency:WordLength + WordPosition:SentencePosition + (1 |Subject) + (1|PassageID) + (1|WordID))

We standardized all continuous predictors in each statistical model to reduce multicollinearity and assessed the variance inflation factor (VIF) to evaluate the multicollinearity among variables. No significant multicollinearity was detected in any of the statistical models (VIF < 10; Kutner et al., 2004). Text difficulty and sentence position were effect-coded to allow the model to estimate the main effects based on the mean differences between the levels, similar to those in an ANOVA.

Reading times were analyzed on the raw scale. This choice was motivated by prior work showing an approximately linear relationship between surprisal (− log probability) and reading time, corresponding to a logarithmic relationship between word predictability and processing difficulty (Shain et al., 2024; Smith & Levy, 2013; Wilcox et al., 2023). We conducted standard residual diagnostics (including residuals-versus-fitted and Q–Q plots). These diagnostics did not reveal systematic structure or nonlinearity that would be expected to meaningfully affect the interpretation of the fixed effects relevant to our research questions.

The models were implemented in the lme4 and lmerTest packages in R (Bates et al., 2015; Kuznetsova et al., 2017). The data and code for analyses are openly available at https://doi.org/10.17605/OSF.IO/8VA9D.

## Results

### Reading accuracy and reading times

Participants in both groups showed high accuracy in answering the comprehension questions of the passages, with no significant differences between groups (Easy texts: *M* = 0.94, *SD* = 0.24; Difficult texts: *M* = 0.92, *SD* = 0.27; *β* = 0.44,* z* = 0.84, *p* = 0.40). Similarly, average word-level reading times, calculated after excluding outliers, first and last words (as detailed in section “Data analysis”), did not differ significantly between groups (Easy texts: *M* = 335 ms, *SD* = 117; Difficult texts: *M* = 345 ms, *SD* = 141; β = 7.78, *t* = 0.38, *p* = 0.71).

To further confirm the comparability of groups, we examined reading times for the 20 shared passages. No significant differences were found in average word reading time (Easy texts: *M* = 336 ms, *SD* = 135; Difficult texts: *M* = 332 ms, *SD* = 115; β = 3.93, *t* = 0.20, *p* = 0.84), suggesting that participants across conditions demonstrated comparable reading performance.

### The influences of text difficulty on surprisal effects

We first examined the main effects of word surprisal across the four computational language models. In all models, words with higher surprisal values elicited significantly longer reading times. Robust effects of word surprisal were found for all models: PCFG (β = 2.04, *t* = 2.27, *p* = 0.023), N-gram (β = 3.73, *t* = 4.05, *p* < 0.001), RNNG (β = 4.17, *t* = 4.56, *p* < 0.001), and Transformer (β = 4.56, *t* = 5.26, *p* < 0.001). In addition, we observed a spillover effect of word surprisal, reflected in longer reading times for words that followed those with higher surprisal values. This effect was consistent in all four models: PCFG (β = 1.91, *t* = 2.22, *p* = 0.027), N-gram (β = 4.16, *t* = 4.50, *p* < 0.001), RNNG (β = 5.11, *t* = 5.61, *p* < 0.001), Transformer (β = 4.82, *t* = 5.51, *p* < 0.001).

Importantly, we observed a significant interaction between text difficulty and word surprisal in the RNNG model: Surprisal effects were stronger in the more difficult texts (RNNG: β = 2.27, *t* = 2.10, *p* = 0.036). In contrast, the N-gram model showed a significant interaction between word surprisal and text difficulty in the spillover region (N-gram: β = 2.33, *t* = 2.18, *p* = 0.029). Although the PCFG and Transformer did not show statistically significant interactions, their estimated spillover effects trended in the same direction as those observed in the N-gram model (PCFG: *β* = 1.74, *t* = 1.62, *p* = 0.106; Transformer: β = 1.92, *t* = 1.81, *p* = 0.071).

For syntactic surprisal, no significant main effects were observed (see Appendix Tables 9, 10, 11, 12, 13, 14, 15, 16 for full statistics). However, syntactic surprisal interacted significantly with text difficulty: syntactic surprisal generated a larger spillover effect in the more difficult texts than in easier texts. This pattern was consistent across all four models (PCFG: β = 2.16, *t* = 2.02, *p* = 0.043; N-gram: β = 2.22, *t* = 2.05, *p* = 0.041; Transformer: β = 2.15, *t* = 1.99, *p* = 0.047), although the effect was marginal for RNNG (β = 2.08, *t* = 1.96, *p* = 0.051) (Tables 6, 7).

**Table 6:** Effects of word surprisal and text difficulty on reading time across language models

Predictors	PCFG		N-gram		RNNG		Transformer	
	β	*p*	β	*p*	β	*p*	β	*p*
WS	2.04	0.023	3.73	< 0.001	4.17	< 0.001	4.56	< 0.001
WS (previous word)	1.91	0.027	4.16	< 0.001	5.11	< 0.001	4.82	< 0.001
TD	4.61	0.822	4.91	0.810	4.88	0.811	4.69	0.819
WS × TD	0.94	0.384	2.01	0.060	2.27	0.036	1.21	0.256
WS (previous word) × TD	1.74	0.106	2.33	0.029	1.85	0.087	1.92	0.071
Marginal/Conditional R2	0.016/0.442		0.016/0.442		0.016/0.442		0.016/0.442	

*WS* word surprisal, *TD* text difficulty

**Table 7:** Effects of syntactic surprisal and text difficulty on reading time across language models

Predictors	PCFG		N-gram		RNNG		Transformer	
	β	*p*	β	*p*	β	*p*	β	*p*
SS	0.31	0.557	0.84	0.118	0.41	0.441	0.47	0.375
SS (previous word)	0.41	0.438	0.70	0.198	0.32	0.546	0.35	0.520
TD	4.22	0.837	4.22	0.837	4.24	0.836	4.23	0.836
SS × TD	0.71	0.502	0.60	0.576	0.96	0.367	0.50	0.640
SS (previous word) × TD	2.16	0.043	2.22	0.040	2.08	0.050	2.15	0.047
Marginal/Conditional R^2^	0.015/0.442		0.016/0.442		0.015/0.442		0.015/0.442	

*SS* syntactic surprisal, *TD* text difficulty

### Contribution of surprisal to model fit

Although word surprisal from all four models showed reliable main effects on reading times, the strength of its interaction with text difficulty varied across models. We therefore conducted an additional analysis to assess the relative contribution of surprisal to overall model fit. Specifically, we compared, for each language model, a mixed-effects model including word surprisal with a baseline model in which the surprisal predictor was omitted (detailed statistics for the baseline models are provided in Appendix Table 17). Including word surprisal significantly improved model fit across all four types of models (PCFG: χ^2^(4) = 15.02, *p* < 0.01; N-gram: χ^2^(4) = 46.27, *p* < 0.001; RNNG: χ^2^(4) = 66.11, *p* < 0.001; Transformer: χ^2^(4) = 69.61, *p* < 0.001). Although the magnitude of the improvement varied numerically across models, as reflected in differences in ΔAIC (PCFG: 7.02; N-gram: 38.26; RNNG: 58.11; Transformer: 61.61), surprisal consistently contributed meaningful explanatory power in every case. These results indicate that surprisal captures a stable component of reading behavior across models, and that variability in interaction effects with text difficulty is unlikely to arise from a lack of robustness in surprisal itself.

## Discussion

Surprisal, which quantifies how well a word aligns with its preceding context, is a valuable computational measure for investigating the reading process. This study adds evidence that the surprisal effects during reading are influenced by the overall difficulty of the text. Across all four computational language models (PCFG, N-gram, RNNG, and Transformer), text difficulty modulated surprisal effects in a consistent direction, with stronger surprisal effects emerging in more difficult texts. This modulation was statistically reliable for syntactic surprisal in three models (PCFG, N-gram, and Transformer) and marginal in the RNNG model. For word surprisal, the modulation was statistically reliable in the N-gram and RNNG models, while the PCFG and Transformer models showed positive trends in the same direction. Because surprisal values were comparable across easy and difficult texts, the observed interaction cannot be attributed to differences in surprisal range between text sets. Instead, reading times increased more sharply with increased surprisal values in the more difficult texts, suggesting that readers rely more heavily on contextual information when processing more demanding texts.

### Text difficulty modulates surprisal effects

Difficult texts typically include more low-frequency words and complex syntactic structures, both of which increase processing demands (Graesser et al., 2004). These challenges require readers to engage in more effortful meaning retrieval, syntactic parsing, and integration, placing greater demands on working memory (Christianson et al., 2006; Ratcliff et al., 2004; Rayner & Duffy, 1986). To manage this cognitive load, readers increasingly rely on broader contextual information to facilitate processing. As a result, surprisal effects are stronger in more difficult texts, reflecting readers’ increased sensitivity to the degree of alignment between a word and its preceding context. In contrast, easier texts tend to consist of more high-frequency words and simpler syntactic constructions, which makes the meanings of individual words and syntactic structures more readily accessible. As a result, readers rely less on contextual information, and the effects of surprisal are correspondingly reduced.

Additional evidence for this adaptive use of context comes from position-related effects found in this study. Specifically, word position and sentence position interacted to significantly influence reading times: readers spent less time on words near the end of a sentence, and this effect was more pronounced in the first sentence of a passage than in the second. These patterns suggest that readers exhibit a slower start at the beginning of a passage, followed by increased reading speed as contextual information accumulates (see Tables 9, 10, 11, 12, 13, 14, 15, 16, 17 in the Appendix for detailed statistics). While surprisal and positional effects both reflect sensitivity to context, they appear to capture different aspects of contextual influence and are associated with distinct ERP components (Chen et al., 2025). For example, word surprisal is associated with the N400 component, reflecting meaning-based processes, whereas position-related effects are not only associated with the N400 but also with earlier components, such as the N170 and P200, which are sensitive to orthographic and phonological processing. Nonetheless, both effects reinforce the idea that readers flexibly engage context in response to increased processing demands.

Text difficulty modulated not only the magnitude of the word surprisal effect on the current word but also its spillover effect on the subsequent word. It also influenced syntactic surprisal, which appeared primarily as a spillover effect. These findings align with prior findings based on the same self-paced reading paradigm that the spillover effect of surprisal is robust (Fernández Monsalve et al., 2012). Similarly, research on word predictability, a concept closely related to surprisal, reported that word predictability significantly influenced the reading times of the subsequent word (Smith & Levy, 2013). These findings support the assumption that reading unfolds as an incremental process in which cognitive load extends across words. The spillover process may be amplified when reading difficult texts, which demand greater cognitive resources to resolve and integrate linguistic information. As a result, the increased effort required to process unexpected, high-surprisal words is more likely to produce residual processing effects that carry over to the following word.

Properties of the self-paced reading paradigm may also contribute to the robust spillover effect observed in our study. Readers control their pace, allowing them to adopt strategies that influence how cognitive resources are allocated. For example, participants might develop a buffering strategy, rapidly advancing to the next word, while postponing the full processing of meaning until more words are read. However, the spillover effect is not unique to self-paced reading. It has also been observed in more natural reading paradigms, including eye-tracking (Berzak & Levy, 2023; Smith & Levy, 2013; Wilcox et al., 2023). Thus, although experimental methods may shape reading behavior, the generality of spillover effects across paradigms suggests that the incrementality in reading is best understood as a set of overlapping phases that extend across more than one word.

To examine whether the modulation of surprisal by text difficulty generalizes across models, we included four computational language models with distinct architectures. For syntactic surprisal, the modulation by text difficulty was statistically reliable in three models (PCFG, N-gram, and Transformer) and marginal in the RNNG model. For word surprisal, the modulation was statistically reliable in the N-gram and RNNG models, while the PCFG and Transformer models showed positive effects in the same direction. Taken together, the modulation was statistically reliable in most cases and converged in direction across all models, suggesting that surprisal captures an important dimension of processing difficulty that generalizes across diverse computational implementations. It is important to note that surprisal estimates derived from different language models are highly correlated, which can potentially complicate the interpretation of statistical significance across multiple closely related analyses. Because the goal of the present study is to evaluate the generalization of the modulation of surprisal by text difficulty, we treat the analyses reported here as robustness checks across correlated predictors rather than as a set of independent hypothesis tests. Interpretation, therefore, emphasizes the consistency of effects across models rather than the significance of any single coefficient. Stronger confirmatory inference would require additional statistical constraints beyond those used here.

It is noteworthy that text difficulty did not show a significant main effect on word-level reading times in any of the statistical models. The absence of the text difficulty effect may reflect the joint influence of multiple factors. First, self-paced reading allows participants to regulate their pace, often leading to a relatively steady pacing that reduces variability across words within a sentence. Such pacing may attenuate the main effect of text difficulty by reducing variability in average reading times. Second, readers may engage predictive and integrative strategies more effectively when reading difficult texts, compensating for higher processing demands and thereby diminishing group-level differences in average reading times. Finally, text difficulty represents an aggregate property that captures variability across multiple linguistic dimensions, including lexical, syntactic, and discourse features. Thus, although it captures overall difficulty, it may be less predictive of reading behavior at the local (word) level.

### Implications of applying surprisal in reading research

Surprisal is a powerful computational framework for linking statistical probability to human reading behavior and is widely used in reading research. Previous studies have emphasized improving the generative accuracy of language models, for example, by selecting language models with appropriate architectures or sizes (Merkx & Frank, 2020; Oh et al., 2022) or by optimizing training datasets (Hale et al., 2019) to enhance how well surprisal captures human reading behavior. Our findings on text difficulty extend this line of work by showing that the characteristics of the texts being read also play a critical role. Readers adapt their processing strategies based on text features; this adaptation can, in turn, directly influence the explanatory power of surprisal in modeling human reading behavior. In the present study, surprisal effects were attenuated when participants read easier texts, suggesting that the predictive/integrative processes reflected by surprisal are less engaged when reading processes are relatively effortless. Although our materials did not include extremely challenging texts, the surprisal effect may also be diminished under conditions of excessive difficulty, where comprehension breakdowns are more likely (Britton et al., 1978). Supporting this possibility, Huang et al. (2024) found that surprisal was a limited predictor in explaining reading behavior for texts containing complex and infrequent constructions (e.g., garden path sentences, object-extracted relative clauses, attachment ambiguities, and subject-verb mismatches).

More broadly, examining surprisal effects requires considering other text features because reading involves multiple interacting processes across different levels of text structure (e.g., word, sentence, and discourse). In this study, we focused specifically on standard measures of text difficulty and used short passages consisting of two sentences, which were sufficient to detect surprisal effects on each word. Although we expect text difficulty to modulate surprisal effects in longer texts, additional factors, such as topic-level coherence or title information, may also come into play and further influence surprisal effects (Helder et al., 2020).

## Conclusion

This study showed that text difficulty modulates the magnitude of the surprisal effect in reading. The surprisal effect is larger during reading more difficult texts, which places higher demands on comprehension, suggesting that readers rely more heavily on the contextual information to support comprehension. These findings support that readers adapt their processing in response to changes in text information.

## Data Availability

All data and code for analyses are available at: (https://doi.org/10.17605/OSF.IO/8VA9D).

## Appendix

**Table:**

Passage numbers	Difficult texts
1	In the right hands, location data can be a force for good. It can provide publishers and app developers with advertising revenue so consumers can access free services and information
2	Paying for college seems out of reach for many Americans, so the idea of free college has broad appeal. Several Democratic presidential candidates and members of Congress have endorsed it
3*	Very few issues can bring together lawmakers of both parties. Animal cruelty is one of them
4*	Koalas have been running into hard times. They have suffered for years from habitat destruction, dog attacks, automobile accidents
5	Some recent reports have suggested that women who give birth by cesarean section are more likely to have obese children. But a large new study found no link between the method of birth and obesity
6*	Pancreatic cancer has a bad reputation. It is a terrible disease, but most people do not realize there are ways that early detection can help
7*	Some nutrition experts say eggs are good for you, even though they are high in cholesterol. Others are sure they are bad
8*	Experts generally agree that watching a lot of television is bad for children. Now a new study suggests it may not be very good for adults, either
9*	Legislators and gun safety advocates often focus on how guns are purchased. But many lives could be saved, especially among children, if they looked more at how they are stored
10	Most cricket species, indoors or outdoors, are omnivorous. They will eat almost any organic material, usually preferring items derived from plants
11	The American medical malpractice system is doing almost nothing to improve the quality of health care, research suggests. What may be more concerning is that there is very little discussion, much less action, on reform
12	Many studies have found that obesity is associated with an increased risk for prostate cancer. Now a new study suggests that the degree of risk may depend on where in the body the fat is
13*	In general, any exercise improves our health. But a wealth of recent science and personal experience indicate that different people can respond to similar exercise routines in different ways
14	A lifelong swimmer leapt into deep water near his lakeside home, and was horrified to find himself completely unable to swim. Had his wife not rescued him, he might have drowned
15	Scientists have used genetically reprogrammed bacteria to destroy tumors in mice. The innovative method one day may lead to cancer therapies that treat the disease more precisely, without the side effects of conventional drugs
16	A cluster of police suicides has prompted leaders to plead with troubled officers to seek help. But resistance is strong
17	Positional vertigo is a common type of dizziness that can be treated with a simple maneuver. Vertigo is an illusory sensation of motion that is often accompanied by intense nausea
18*	When new breast cancer drugs come to market, there is little data to indicate whether they are safe or effective in men. Some new drugs are approved only for women
19	Polio is an infectious disease that can spread rapidly and mainly affects young children. It can cause muscle weakness, paralysis and, on rare occasions, it can be fatal
20	In general, mammalian hearts are quite malleable. They change in response to the demands placed on them
21	A number of countries, including Australia and Germany, have started requiring companies to report their gender pay gaps in recent years. Proponents argue that transparency can lead to improvement
22*	An extensive study confirms that red meat might not be that bad for you. But it is bad for the planet, with chicken and pork less harmful than beef
23	The caterpillar of the monarch butterfly eats only milkweed, a poisonous plant that should kill it. The caterpillars thrive on the plant, even storing its toxins in their bodies as a defense against hungry birds
24	A decline in income in young adulthood may lead to poorer cognitive ability in middle age. Income reductions are also linked to changes in brain structure that suggest premature aging, researchers report
25	Many patients with the mysterious illness have wound up hospitalized in intensive care units, needing ventilators or even more desperate measures to help them breathe. Most are young, male adults or even teenagers
26*	The ground around the building slopes away. That allows the classroom windows to be low enough for children to see out while still being high above the ground
27	A surge of oil production is coming, whether the world needs it or not. The flood of crude will arrive even as concerns about climate change are growing and worldwide oil demand is slowing
28*	Farmers and plant breeders are in a race against time. The world population is growing rapidly, requiring ever more food, but the amount of cultivable land is limited
29	Two young black women face futures made precarious by sudden violence wrought by men in their lives. One is a shy Florida high schooler struggling to find solace as her family tears itself apart
30*	One benefit of discovery is that you get to name the things you discovered. Astronomy is blessed in this regard
31	On bright mornings in the tropics, crocodiles haul themselves out of the water to bask in the sun. Like other reptiles, they rely on its warmth to help fuel their metabolisms
32	Elephants keep surprising us. They live complex social lives, cooperate, show altruism and grieve their dead
33*	The overwhelming majority of snails live in shells that coil to the right. But occasionally some are born with shells that turn the other way
34	To visit Venezuela today is to see how misrule can shatter a country. Years of corrupt, incompetent and autocratic rule have left Venezuelans hungry and children dying for want of basic medical care
35*	California has positioned itself as a leader in the fight against climate change. It leads the nation by far in solar power
36	This has been a golden age for brain research. We now have amazing brain scans that show which networks in the brain ramp up during different activities
37	Student debt is spiraling, and young people are defaulting on their loans. You might think that students would be better off scraping by with no loans at all
38	The bombs smashed into a child care center, a refugee camp and a school. They destroyed makeshift clinics and hospitals, disabling essential services for tens of thousands of people
39	To make it to the big game, the Saudi soccer fan resorted to unusual measures. He first flew to Kuwait, where he told his parents he was going on vacation
40	It was bound to happen. Toothpaste, perhaps the last part of your daily grooming regimen that had not been gussied up, has now been rethought
41	Done correctly, exfoliation helps your skin purge dead cells, revealing a better complexion underneath. That magic draws us to the practice but also makes us overzealous
42	By the time most military children complete secondary school, they will have switched schools up to nine times. These frequent changes between schools of strikingly varying quality trouble military children both socially and academically
43	A decade ago, natural gas was heralded as the fuel of the future. In shale fields across the country, hydraulic fracturing uncorked a lucrative new source of supply
44*	Climate change will inevitably transform the way the world produces goods. Farmers who produce wine grapes, an especially sensitive crop, are already feeling those effects
45	Progressives have long looked to federal courts to guard the rights of racial minorities and dissenters. But that protection is weakening
46	Ten years ago, checklists for surgeons were all the rage. Inspired by the preflight routines of airline pilots, surgical checklists were shown to prevent tragic errors, reduce infections and save lives
47*	The many whiskeys of Japan are complex and often expensive. Learning about them can be a challenge
48	Dublin has long been a beer and whiskey town. For generations pubs poured the same pints of stout and familiar bottles of whiskey, but in recent years a shift has shaken the local drinking scene
49	For airlines, time on the ground is money. Financially speaking, planes only earn revenue for their companies when they are in the air, ferrying paying passengers
50	Traveling can wreak havoc on your fitness. If you are lucky, all the sightseeing you do on foot will cancel out the nighttime eating and drinking
51	Even if you strive for a sustainable lifestyle at home, it may be tempting to avoid thinking about the impact your travels could have on the environment. No one wants to feel guilty on vacation
52*	Great snow conditions can make a ski trip magical. Skiers can weight the odds in their favor by picking their destinations according to the time of winter
53	Tent camping should be one of the cheapest forms of travel, subject to nominal campsite and park admission fees. But, short of borrowing gear, bringing your own bedroll and shelter can be costly
54	Family separations at the border are not the only crisis immigrant children face. Children who were taken from their parents, or who crossed the border alone, also go to their immigration hearings alone
55	Winning civil rights is never easy. The fight can stretch on for decades and include setbacks that feel like utter defeat
56	For the rural firefighters of Australia, no donation is too small. They have long been known to canvass for dollars and cents in town markets, and their collection tins are fondly displayed in local storefronts
57	Our relationship to privacy is inseparable from our idea of trust. If you trust someone, you may be more willing to divulge personal information to them
58	Businesses do themselves a disservice by shying away from older workers. Rather than worrying about only the potential cost of a more experienced worker, hiring managers should also consider the benefits of hiring them
59*	Earth is warming, and we know why. Light is reflected and absorbed by clouds, air, oceans, ice and land
60*	Nobody ever really tells you how to breathe. You just know how to do it
61	The immense wall of crumbling, whitewashed stone might have belonged to an abandoned castle. Turrets lined a roof that was no longer there, and watchtowers poked the sky at each corner
62	You never know where you will find love. Even on a crowded airplane or a cruise ship
63*	Imagine an animal that looks like a dinosaur, and you probably will not imagine a bat. But that may change

The passages marked with an asterisk (*) are shared ones.

**Table:**

Passage numbers	Easy texts
101*	Very few issues can bring together lawmakers of both parties. Animal cruelty is one of them
102*	Koalas have been running into hard times. They have suffered for years from habitat destruction, dog attacks, automobile accidents
103*	Pancreatic cancer has a bad reputation. It is a terrible disease, but most people do not realize there are ways that early detection can help
104*	Some nutrition experts say eggs are good for you, even though they are high in cholesterol. Others are sure they are bad
105*	Experts generally agree that watching a lot of television is bad for children. Now a new study suggests it may not be very good for adults, either
106*	Legislators and gun safety advocates often focus on how guns are purchased. But many lives could be saved, especially among children, if they looked more at how they are stored
107*	In general, any exercise improves our health. But a wealth of recent science and personal experience indicate that different people can respond to similar exercise routines in different ways
108*	When new breast cancer drugs come to market, there is little data to indicate whether they are safe or effective in men. Some new drugs are approved only for women
109*	An extensive study confirms that red meat might not be that bad for you. But it is bad for the planet, with chicken and pork less harmful than beef
110*	The ground around the building slopes away. That allows the classroom windows to be low enough for children to see out while still being high above the ground
111*	Farmers and plant breeders are in a race against time. The world population is growing rapidly, requiring ever more food, but the amount of cultivable land is limited
112*	One benefit of discovery is that you get to name the things you discovered. Astronomy is blessed in this regard
113*	The overwhelming majority of snails live in shells that coil to the right. But occasionally some are born with shells that turn the other way
114*	California has positioned itself as a leader in the fight against climate change. It leads the nation by far in solar power
115*	Climate change will inevitably transform the way the world produces goods. Farmers who produce wine grapes, an especially sensitive crop, are already feeling those effects
116*	The many whiskeys of Japan are complex and often expensive. Learning about them can be a challenge
117*	Great snow conditions can make a ski trip magical. Skiers can weight the odds in their favor by picking their destinations according to the time of winter
118*	Earth is warming, and we know why. Light is reflected and absorbed by clouds, air, oceans, ice and land
119*	Nobody ever really tells you how to breathe. You just know how to do it
120*	Imagine an animal that looks like a dinosaur, and you probably will not imagine a bat. But that may change
121	Video games work hard to hook players. Designers use predictive algorithms and principles of behavioral economics to keep fans engaged
122	Americans are feeling better about the economy. Or at least Republicans are
123	Parasites are a huge force in the natural world. For the most part they simply feed on their hosts
124	There should be no question that autistic people need and value social connections. We all do
125	Once upon a time, the sky was everything above the earth, which lay at the center of human experience. The sky was the place of gods
126	The abundance of uncontrolled case studies can give an impression that a single dietary item, like juice, is linked with obesity. But they cannot determine cause and effect
127	Americans are having fewer babies. At first, researchers thought the declining fertility rate was because of the recession, but it kept falling even as the economy recovered
128	New research suggests that more money really does lead to a more satisfying life. Surveys of thousands of Swedish lottery winners have provided persuasive evidence of this truth
129	Milan has no sea. It has no river or lake, just a few ebbing canals
130	Morality exists only because we evolved the capacity to empathize. And empathy provides the best guide to moral action
131	Story creates culture. It teaches us to feel, think and behave in ways generally approved of by those around us
132	Hospitals are often thought of as the hubs of our health care system. But hospital closings are rising, particularly in some types of communities
133	Two sexes have never been enough to describe human variety. Not in biblical times and not now
134	The biggest racial preferences in this country have nothing to do with college admissions or job offers. They have to do with political power
135	Businesses can deliver everything on demand, from dinner to dry cleaning. Some will even show up at your door to give you cupcakes or walk your dog
136	Planning for vacation is fun. We make lists of clothes to pack and museums to visit, anticipating the relaxation and fun to come
137	Certain types of wine seem incapable of winning popular acceptance. Riesling is one, particularly in its moderately sweet form
138	Bankers kept their name tags obscured behind ties. Many tried to keep a low profile and avoided talking to the news media
139	Hollywood executives are not so different from the stars they employ. They want to be seen in the right booths at the right restaurants
140	The more we learn about how people really think, the more we must rethink economic theory. Changes in fundamental beliefs play a major role in the fluctuations of the economy
141	History suggests that the world is about due for another financial crisis. One of the places it might start, according to a growing number of indicators, is Italy
142	Few doubt that energy has improved lives and enabled human progress. Yet one of the biggest challenges facing the world is the polarized debate over the future of energy
143	Dance is about change. The body keeps altering its shape while we watch it move
144	Plants have no eyes, no ears, no mouth and no hands. They do not have a brain or a nervous system
145	There are many more gorillas and chimpanzees than previously believed, new research finds. Nonetheless, their numbers are rapidly declining
146	Forests are disappearing. Maps show shrinking woodlands all over the world
147	A cowboy walks into a bar. Or more specifically, a Nigerian immigrant grad student in a cowboy hat walks into a bar in Oklahoma
148	One man vanished diving at midnight. Another was attacked by a great white shark in deep water
149	Student loans are central to financing college educations, yet millions of borrowers are in default. That is clear evidence that the system is in dire need of improvement
150	People seem to be more worried than ever about stress. We hear that stress can lead to everything from depression to cancer
151	Music can transport us back in time or help usher in new ways of thinking. It can capture a historical moment or offer an escape from the world around us
152	The laws that authorize medical aid in dying have worked exactly as intended, the evidence shows. They have benefited dying patients and their families without causing any harm to anyone
153	Israel cannot define a Jew. And the more it attempts to do so, the more obvious that becomes
154	Corporate tax cuts will put billions of dollars back in the hands of businesses this year. Naturally, people want to know how those businesses will spend it
155	Florida, it seems, has always been a popular destination. Even the first known Americans gravitated to the state
156	When early humans discovered how to build fires, life became much easier in many regards. They huddled around fire for warmth, light and protection
157	For most of human history, nobody ate a vegetable for pleasure. After all, vegetables have no evolutionary imperative to be delicious
158	Animals around the world are on the move. So are their parasites
159	Gifts are often left on front row seats at fashion shows for guests. Rarely, however, are they placed on every seat
160	Trying to figure out why humans cry is exhausting. We cry about death, violence, breakups, abandoned puppies, sweet kisses and words charged with all kinds of meanings
161	The most popular movie in India this summer is about a toilet. It nearly causes a divorce
162	Bargain hunters have begun to pay close attention to emerging market stocks. They are a little late to the game
163	One new drug promises to stop cancer from spreading to other organs. Another would treat blood cancer

See Tables 8, 9, 10, 11, 12, 13, 14, 15, 16 and 17.

**Table 8:** Kolmogorov–Smirnov test results for surprisal distributions between easy and difficult texts

Surprisal measure	D statistic (maximum difference between the cumulative distribution functions)	*p* value
PCFG Word Surprisal	0.05	0.14
PCFG Syntactic Surprisal	0.04	0.47
N-gram Word Surprisal	0.04	0.27
N-gram Syntactic Surprisal	0.04	0.43
RNNG Word Surprisal	0.06	0.045
RNNG Syntactic Surprisal	0.03	0.55
Transformer Word Surprisal	0.06	0.045
Transformer Syntactic Surprisal	0.02	0.95

**Table 9:** PCFG word surprisal model

*Predictors*	RT		
	*Estimates*	*CI*	*p*
(Intercept)	337.65	317.56–357.74	**< 0.001**
WS	2.04	0.28–3.79	**0.023**
WS (previous word)	1.91	0.22–3.60	**0.027**
TD (EasyText Vs. DifficultText)	4.61	− 35.52–44.74	0.822
WF	− 3.61	− 5.89 to − 1.33	**0.002**
WL	4.93	3.00–6.87	**< 0.001**
WF (previous word)	− 6.49	− 8.75 to − 4.23	**< 0.001**
WL (previous word)	4.73	2.89–6.57	**< 0.001**
WP	− 1.01	− 2.18–0.16	0.092
Sentence1 Vs. Sentence2	− 2.10	− 4.25–0.04	0.055
WS × TD (EasyText Vs. DifficultText)	0.94	− 1.18–3.07	0.384
WS (previous word) × TD (EasyText Vs. DifficultText)	1.74	− 0.37–3.85	0.106
WF × WL	− 2.63	− 3.84 to − 1.41	**< 0.001**
WP × SentenceSentence1Vs Sentence2	3.61	1.22–6.01	**0.003**
Random effects			
σ^2^	9539.05		
τ_00_ _WordNum_	176.52		
τ_00_ _Passage_	91.92		
τ_00_ _Subject_	7026.05		
ICC	0.43		
N_Subject_	68		
N_Passage_	86		
N_WordNum_	1846		
Observations	57,315		
Marginal R^2^/Conditional R^2^	0.016/0.442		

*WS* word surprisal, *TD* text difficulty, *WF* word frequency, *WL* word length, *WP* word position, *SP* sentence position

**Table 10:** PCFG syntactic surprisal model

*Predictors*	RT		
	*Estimates*	*CI*	*p*
(Intercept)	338.00	317.92–358.09	**< 0.001**
SS	0.31	− 0.73–1.35	0.557
SS (previous word)	0.41	− 0.63–1.46	0.438
TD (EasyText Vs. DifficultText)	4.22	− 35.91–44.34	0.837
WF	− 5.39	− 7.23 to − 3.55	**< 0.001**
WL	5.00	3.07–6.93	**< 0.001**
WF (previous word)	− 8.28	− 10.12 to − 6.44	**< 0.001**
WL (previous word)	4.68	2.84–6.52	**< 0.001**
WP	− 1.13	− 2.31–0.05	0.061
Sentence1 Vs. Sentence2	− 2.08	− 4.23–0.08	0.059
SS × TD (EasyText Vs. DifficultText)	0.71	− 1.36–2.77	0.502
SS (previous word) × TD (EasyText Vs. DifficultText)	2.16	0.07–4.25	**0.043**
WF × WL	− 2.30	− 3.48 to − 1.12	**< 0.001**
WP × SentenceSentence1Vs Sentence2	3.60	1.20–6.01	**0.003**
Random effects			
σ^2^	9539.04		
τ_00_ _WordNum_	178.93		
τ_00_ _Passage_	92.54		
τ_00_ _Subject_	7024.88		
ICC	0.43		
N_Subject_	68		
N_Passage_	86		
N _WordNum_	1846		
Observations	57,315		
Marginal R^2^/Conditional R^2^	0.015/0.442		

*SS* syntactic surprisal, *TD* text difficulty, *WF* word frequency, *WL* word length, *WP* word position, *SP* sentence position

**Table 11:** N-gram word surprisal model

*Predictors*	RT		
	*Estimates*	*CI*	*p*
(Intercept)	337.65	317.57–357.73	**< 0.001**
WS	3.73	1.92–5.53	**< 0.001**
WS (previous word)	4.16	2.35–5.97	**< 0.001**
TD (EasyText Vs. DifficultText)	4.91	− 35.21–45.04	0.810
WF	− 1.86	− 4.21–0.48	0.120
WL	5.17	3.25–7.08	**< 0.001**
WF (previous word)	− 4.49	− 6.83 to − 2.14	**< 0.001**
WL (previous word)	4.60	2.78–6.42	**< 0.001**
WP	− 0.89	− 2.05–0.27	0.134
Sentence1 Vs. Sentence2	− 1.94	− 4.06–0.19	0.074
WS × TD (EasyText Vs. DifficultText)	2.01	− 0.09–4.11	0.060
WS (previous word) × TD (EasyText Vs. DifficultText)	2.33	0.24–4.42	**0.029**
WF × WL	− 2.54	−3.72 to −1.37	**< 0.001**
WP × SentenceSentence1Vs Sentence2	3.85	1.47–6.22	**0.002**
Random effects			
σ^2^	9539.18		
τ_00_ _WordNum_	168.18		
τ_00_ _Passage_	90.30		
τ_00_ _Subject_	7025.20		
ICC	0.43		
N_Subject_	68		
N_Passage_	86		
N_WordNum_	1846		
Observations	57,315		
Marginal R^2^/Conditional R^2^	0.016/0.442		

*WS* word surprisal, *TD* text difficulty, *WF* word frequency, *WL* word length, *WP* word position, *SP* sentence position

**Table 12:** N-gram syntactic surprisal model

*Predictors*	RT		
	*Estimates*	*CI*	*p*
(Intercept)	337.96	317.88–358.05	**< 0.001**
SS	0.84	− 0.21–1.89	0.118
SS (previous word)	0.70	− 0.36–1.76	0.198
TD (EasyText Vs. DifficultText)	4.22	− 35.91–44.35	0.837
WF	− 5.44	− 7.27 to − 3.60	**< 0.001**
WL	4.92	2.99–6.85	**< 0.001**
WF (previous word)	− 8.21	− 10.05 to − 6.36	**< 0.001**
WL (previous word)	4.69	2.84–6.53	**< 0.001**
WP	− 1.10	− 2.28–0.08	0.067
Sentence1 Vs. Sentence2	− 2.04	− 4.18– 0.11	0.063
SS × TD (EasyText Vs. DifficultText)	0.60	− 1.49–2.69	0.576
SS (previous word) × TD (EasyText Vs. DifficultText)	2.22	0.10–4.33	**0.040**
WF × WL	− 2.32	− 3.50 to − 1.14	**< 0.001**
WP × SentenceSentence1Vs Sentence2	3.73	1.33–6.14	**0.002**
Random effects			
σ^2^	9538.94		
τ_00_ _WordNum_	177.90		
τ_00_ _Passage_	92.90		
τ_00_ _Subject_	7025.70		
ICC	0.43		
N_Subject_	68		
N_Passage_	86		
N_WordNum_	1846		
Observations	57,315		
Marginal R^2^/Conditional R^2^	0.016/0.442		

*SS* syntactic surprisal, *TD* text difficulty, *WF* word frequency, *WL* word length, *WP* word position, *SP* sentence position

**Table 13:** RNNG word surprisal model

*Predictors*	RT		
	*Estimates*	*CI*	*p*
(Intercept)	338.05	317.97–358.13	**< 0.001**
WS	4.17	2.38–5.97	**< 0.001**
WS (previous word)	5.11	3.32–6.90	**< 0.001**
TD (EasyText Vs. DifficultText)	4.88	− 35.24–45.00	0.811
WF	− 1.26	− 3.63–1.11	0.297
WL	5.54	3.64–7.45	**< 0.001**
WF (previous word)	− 3.65	− 6.00 to − 1.31	**0.002**
WL (previous word)	4.87	3.05–6.68	**< 0.001**
WP	− 0.95	− 0.11–0.20	0.106
Sentence1 Vs. Sentence2	− 1.92	− 4.03–0.20	0.076
WS × TD (EasyText Vs. DifficultText)	2.27	0.15–4.39	**0.036**
WS (previous word) × TD (EasyText Vs. DifficultText)	1.85	− 0.27–3.96	0.087
WF × WL	− 2.06	− 3.23 to − 0.89	**0.001**
WP × SentenceSentence1Vs Sentence2	3.81	1.44–6.18	**0.002**
Random effects			
σ^2^	9538.87		
τ_00_ _WordNum_	163.58		
τ_00_ _Passage_	89.19		
τ_00_ _Subject_	7025.21		
ICC	0.43		
N_Subject_	68		
N_Passage_	86		
N_WordNum_	1846		
Observations	57,315		
Marginal R^2^/Conditional R^2^	0.016/0.442		

*WS* word surprisal, *TD* text difficulty, *WF* word frequency, *WL* word length, *WP* word position, *SP* sentence position

**Table 14:** RNNG syntactic surprisal model

*Predictors*	RT		
	*Estimates*	*CI*	*p*
(Intercept)	338.04	317.95–358.12	**< 0.001**
SS	0.41	0.63–1.45	0.441
SS (previous word)	0.32	− 0.73–1.37	0.546
TD (EasyText Vs. DifficultText)	4.24	− 35.89–44.37	0.836
WF	− 5.42	− 7.26 to − 3.59	**< 0.001**
WL	4.97	3.04–6.90	**< 0.001**
WF (previous word)	− 8.22	− 10.07 to − 6.38	**< 0.001**
WL (previous word)	4.74	2.89–6.58	**< 0.001**
WP	− 1.12	− 2.30–0.06	0.063
Sentence1 Vs. Sentence2	− 2.06	− 4.21–0.09	0.061
SS × TD (EasyText Vs. DifficultText)	0.96	− 1.12–3.03	0.367
SS (previous word) × TD (EasyText Vs. DifficultText)	2.08	− 0.00–4.17	0.050
WF × WL	− 2.29	− 3.47 to − 1.12	**< 0.001**
WP × SentenceSentence1Vs Sentence2	3.65	1.24–6.06	**0.003**
Random effects			
σ^2^	9538.97		
τ_00_ _WordNum_	178.90		
τ_00_ _Passage_	93.44		
τ_00_ _Subject_	7025.55		
ICC	0.43		
N_Subject_	68		
N_Passage_	86		
N_WordNum_	1846		
Observations	57,315		
Marginal R^2^/Conditional R^2^	0.015/0.442		

*SS* syntactic surprisal, *TD* text difficulty, *WF* word frequency, *WL* word length, *WP* word position, *SP* sentence position

**Table 15:** Transformer word surprisal model

*Predictors*	RT		
	*Estimates*	*CI*	*p*
(Intercept)	337.74	317.66–357.82	**< 0.001**
WS	4.56	2.86–6.26	**< 0.001**
WS (previous word)	4.82	3.10–6.53	**< 0.001**
TD (EasyText Vs. DifficultText)	4.69	− 35.43–44.80	0.819
WF	− 1.11	− 3.40–1.18	0.341
WL	5.42	3.51–7.32	**< 0.001**
WF (previous word)	− 3.72	− 6.01 to − 1.44	**0.001**
WL (previous word)	4.97	3.16–6.78	**< 0.001**
WP	− 0.89	− 2.05–0.26	0.129
Sentence1 Vs. Sentence2	1.90	− 4.01–0.21	0.078
WS × TD (EasyText Vs. DifficultText)	1.21	− 0.88–3.30	0.256
WS (previous word) × TD (EasyText Vs. DifficultText)	1.92	− 0.16–4.01	0.071
WF × WL	− 2.49	− 3.66 to − 1.33	**< 0.001**
WP × SentenceSentence1Vs Sentence2	3.58	1.21–5.94	**0.003**
Random effects			
σ^2^	9539.16		
τ_00_ _WordNum_	162.34		
τ_00_ _Passage_	88.44		
τ_00_ _Subject_	7025.00		
ICC	0.43		
N_Subject_	68		
N_Passage_	86		
N_WordNum_	1846		
Observations	57,315		
Marginal R^2^/Conditional R^2^	0.016/0.442		

*WS* word surprisal, *TD* text difficulty, *WF* word frequency, *WL* word length, *WP* word position, *SP* sentence position

**Table 16:** Transformer syntactic surprisal model

*Predictors*	RT		
	*Estimates*	*CI*	*p*
(Intercept)	338.01	317.92–358.10	**< 0.001**
SS	0.47	− 0.57–1.52	0.375
SS (previous word)	0.35	− 0.71–1.41	0.520
TD (EasyText Vs. DifficultText)	4.23	− 35.90–44.36	0.836
WF	− 5.38	− 7.22 to − 3.54	**< 0.001**
WL	5.01	3.08–6.94	**< 0.001**
WF (previous word)	− 8.23	− 10.08 to − 6.39	**< 0.001**
WL (previous word)	4.73	2.88–6.57	**< 0.001**
WP	− 1.13	− 2.31–0.05	0.061
Sentence1 Vs. Sentence2	− 2.06	− 4.21–0.09	0.061
SS × TD (EasyText Vs. DifficultText)	0.50	− 1.59–2.59	0.640
SS (previous word) × TD (EasyText Vs. DifficultText)	2.15	0.03–4.26	**0.047**
WF × WL	− 2.29	− 3.47 to − 1.11	**< 0.001**
WP × SentenceSentence1Vs Sentence2	3.69	1.28–6.10	**0.003**
Random effects			
σ^2^	9539.00		
τ_00_ _WordNum_	178.89		
τ_00_ _Passage_	93.16		
τ_00_ _Subject_	7025.64		
ICC	0.43		
N_Subject_	68		
N_Passage_	86		
N_WordNum_	1846		
Observations	57,315		
Marginal R^2^/Conditional R^2^	0.015/0.442		

*SS* syntactic surprisal, *TD* text difficulty, *WF* word frequency, *WL* word length, *WP* word position, *SP* sentence position

**Table 17:** Baseline model

*Predictors*	RT		
	*Estimates*	*CI*	*p*
(Intercept)	337.98	317.90–358.07	**< 0.001**
TD (EasyText Vs. DifficultText)	4.23	− 35.90–44.36	0.836
WF	− 5.35	− 7.19 to − 3.51	**< 0.001**
WL	5.05	3.12–6.98	**< 0.001**
WF (previous word)	− 8.28	−10.11 to −6.44	**< 0.001**
WL (previous word)	4.76	2.92–6.60	**< 0.001**
WP	− 1.11	− 2.28–0.07	0.065
Sentence1 Vs. Sentence2	− 1.92	− 4.07– 0.23	0.080
WF × WL	− 2.30	− 3.48 to − 1.12	**< 0.001**
WP × SentenceSentence1Vs Sentence2	3.65	1.25–6.06	**0.003**
Random effects			
σ^2^	9539.01		
τ_00_ _WordNum_	180.45		
τ_00_ _Passage_	93.29		
τ_00_ _Subject_	7025.38		
ICC	0.43		
N_Subject_	68		
N_Passage_	86		
N_WordNum_	1846		
Observations	57,315		
Marginal R^2^/Conditional R^2^	0.015/0.442		

*TD* text difficulty, *WF* word frequency, *WL* word length, *WP* word position, *SP* sentence position
